# Molecular Identification and Evolutionary Divergence of the Sri Lankan Sambar Deer, *Rusa unicolor* (Kerr 1792)

**DOI:** 10.3390/ani13182877

**Published:** 2023-09-10

**Authors:** Subodha Lakruwani Jayasundara, Hirusha Randimal Algewatta, Suhada Jayawardana, Minoli Perera, L. Dinithi C. Peiris

**Affiliations:** 1 Department of Zoology, Faculty of Applied Sciences, University of Sri Jayewardenepura, Nugegoda 10250, Sri Lanka; subodhajayasundara1@gmail.com (S.L.J.); mperera95826@gmail.com (M.P.); 2 Wildlife Rehabilitation Center, Department of Wildlife Conservation, 811A, Jayanthipura, Btataramulla 10120, Sri Lanka; suhadawildlifevet@gmail.com; 3 Genetics & Molecular Biology Unit/Department of Zoology, Faculty of Applied Sciences, University of Sri Jayewardenepura, Nugegoda 10250, Sri Lanka

**Keywords:** *Rusa unicolor*, molecular identification, *Cytochrome oxidase subunit I (COI)*, *Cytochrome b (Cyt b)*, phylogeny, Sambar deer, taxonomy, evolutionary divergence

## Abstract

**Simple Summary:**

*Rusa unicolor* (the Sambar deer) has been listed as a vulnerable species by the International Union for Conservation of Nature and Natural Resources (IUCN). These mammals play a significant role in the region’s biodiversity and are vital to the ecological system’s well-being. Because of their remarkable physical resemblances, differentiating between Sambar deer species can be challenging, often resulting in overlap across classifications. Hence, as a substitute for solely relying on morphological identification, a DNA barcode system can be employed as the best solution to address the challenges linked to their taxonomic classification. For the first time, our findings unveil that the Sri Lankan Sambar deer constitutes a distinct genetic subspecies. The outcome offers important molecular information, setting the foundation for future efforts in the conservation of these species.

**Abstract:**

The Sambar is one of the largest deer species distributed mainly in Asia, and it has been listed as a vulnerable species. Taxonomy based on morphological characterization has been the gold standard method used to identify the Sambar deer species. Yet, morphological identification is challenging and requires expertise. To conduct species identification and taxonomic decisions, we performed the molecular identification of *R. unicolor* found in Sri Lanka using DNA barcodes, *COI*, and *Cyt b* to compare the Sri Lankan *R. unicolor* with the Indian *R. unicolor* and other *R. unicolor* subspecies. We obtained mitochondrial DNA sequences from *COI* and *Cyt b* from blood samples collected from the wet zone in Sri Lanka. A phylogenetic tree was constructed based on the Bayesian analyses using MrBayes 3.2.7. Molecular dating was implemented in Bayesian Evolutionary Analysis Sampling Trees (BEAST v1.8.2) on the concatenated sequence using a log-normal relaxed clock and Yule species tree prior, with four categories. The results showed that the Sri Lankan *R. unicolor* is genetically different from the Indian *R. unicolor* and other *R. unicolor* subspecies. The divergence occurred approximately 1.1 MYA (million years ago) in the Pleistocene era. The results are essential for designing new conservation platforms for these Sambar deer species.

## 1. Introduction

The *Rusa unicolor* is the largest oriental deer (common names: the Sambar deer in English and “Gona” in Sinhala) belonging to the order Artiodactyla, family Cervidae, and subfamily Cervinae. In mammals, there are two ungulate orders: Perissodactyla and Artiodactyla. The most common means of recognizing the two types of ungulates are based on their hooves. Perissodactyls possess an odd number of toes, while Artiodactyls have several, and they appear in even numbers. Sambar deer have grey-brown skin covered by dark brown hair, which can also appear slightly reddish and darker along its midline. They mainly have blackish bushy tails and whitish undersides and rump areas. The male Sambar is darker than the female Sambar and has long, aspirate hair on its abdomen, neck, and back. It can weigh up to 320 kg and measure two meters in height from head to tail. The antlers of mature males are unique among cervids. 

Sambar antlers have three to four tines that are shed and replaced periodically [1]. This is the most widespread deer species in Asia [2]. They can be found in Southern China [1,3], Sri Lanka [1], India, and Burma and throughout Southern Nepal [4,5,6] and Southeast Asia to the Pacific Coast, as well as on the islands of Borneo, Hainan, and Taiwan. The body masses, colors, and antler lengths of Sambar deer vary among the subspecies of *Rusa*, and the variability increases regionally from east to west with their distribution [1,7]. There are seven recognized subspecies of *R. unicolor*: *Rusa unicolor brookei*, *R. u. cambojensis*, *R. u. dejeani*, *R. u. equinus*, *R. u. swinhoei*, *R. u. hainana*, and *R. u. unicolor* [8].

Sambar deer are primarily nocturnal mammals that utilize open grassland and scrub areas. They prefer forested regions with minimal human disturbance that have free access to water. They have developed predominately crepuscular and nocturnal activities in response to human hunting and predatory pressures. They are typically solitary and can be spotted in small groups during their breeding seasons. Sambars feed on leaves, berries, grasses, bark from young trees, fallen fruit, herbs, and buds [1]. With recent human and environmental conflicts, Sambar deer have been confined to the Wilpattu, Ritigala, Wasgamuwa, Knuckles range, Haggala, Horton plains, Udawalawe, Yala, and Kumana regions of Sri Lanka [9]. *R. unicolor* has been listed as a vulnerable species by the IUCN because of predation, overexploitation for subsistence, and demands for its meat and antlers [10]. Considerable attention has been gained regarding the alarming decline in Sambar deer species. Hence, conservation pursuits are being undertaken by various authorities worldwide. 

Genetic data offer significant value in the conservation of animals that are in critical situations. Mitochondrial DNA (mtDNA) is commonly employed in conservation genetics due to its cost-efficient approach in determining the genetic makeup of species that have not been studied before. mtDNA evolves more rapidly than genomic DNA, accumulating sequence variations between closely related species [11]. Therefore, mtDNA is a highly effective resource in studying animal groups’ population genetics and evolutionary paths. mtDNA is more abundant in cells than nuclear DNA, and the mutation rate for mtDNA is 5–10 times greater than that of nuclear DNA, leading to accumulation of sequence variations between species [12]. Though several mammalian mtDNA genomes have been sequenced, within the seven subspecies of *Rusa unicolor*, only the complete mitochondrial genomes of *R. u. swinhoei*, *R. u. dejeani*, *R. u. hainana,* and *R. u. cambojensis* are available at present [13]. However, more studies and tools on Sambar deer taxonomy are essential for accurate identification. 

Under molecular identification, DNA barcoding is a standardized approach for identifying animals and plants with minimal DNA sequences (called DNA barcodes). The introduction of the DNA barcoding approach has become the gold standard used in taxonomic studies. DNA barcoding has been used for cervid identification in India [14] and Southwest China [15]. A previous study conducted in Kerala, India, using *COI* barcode sequences, revealed that the Indian Sambar deer has faced continuous poaching by illegal hunters. Hence, DNA barcoding is essential in wildlife forensics [16]. Though several studies have been carried out in this Asian region using this DNA barcoding to resolve the phylogeny of *Rusa unicolor,* studies for identifying *Rusa unicolor* in Sri Lanka are scarce. 

Therefore, in the present study, the *Cytochrome b* (*Cyt b*) and *Cytochrome Oxidase subunit one* (*COI*) genes were sequenced and analyzed to smooth the path towards *R. unicolor* identification in Sri Lanka and to provide fundamental molecular data for the further conservation of these large mammals. Possible relationships between Indian *R. unicolor* species are also discussed. This study showed that the Sri Lankan *R. unicolor* has genetic divergence from the Indian *R. unicolor* and other *R. unicolor* subspecies. Molecular taxonomy can help to resolve phylogenetic relationships among species for effective conservation planning.

## 2. Materials and Methods

### 2.1. Sample Collection

**Figure 1:**
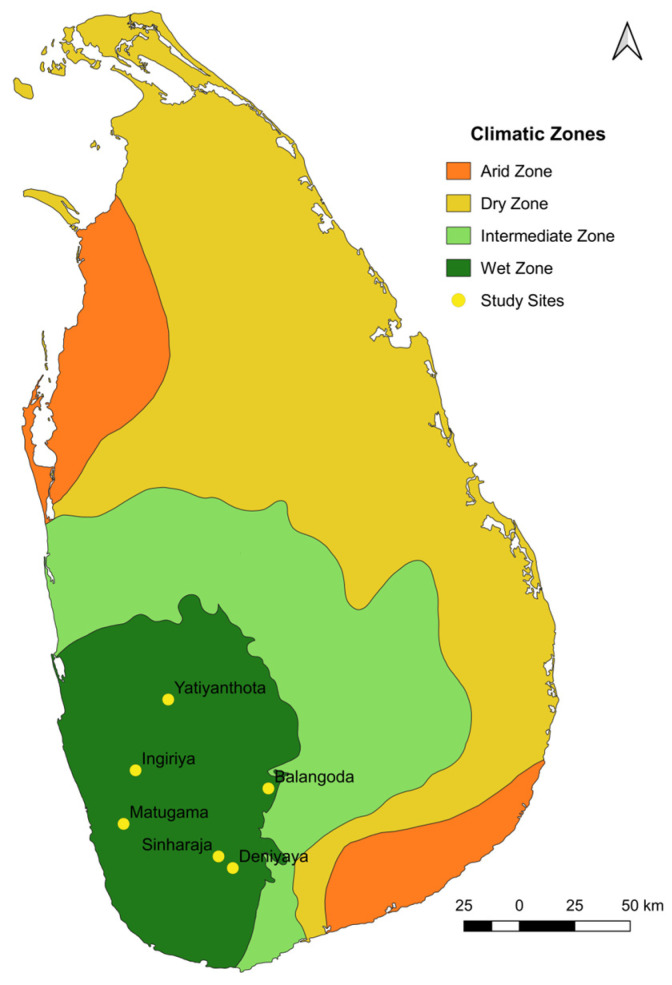
Map of the collection sites in the wet zone region. The collection sites in the wet zone of Sri Lanka are marked with yellow dots. The map was constructed using GPS tracking data.

The study was carried out in the wet zone of Sri Lanka as follows: Ingiriya (6°44′25.36″ N 80°9′44.83″ E), Matugama (6°31′19″ N 80°6′49.27″ E), Yatiyanthota (7°1′43.98″ N 80°17′43.95″ E), Balangoda (6°40′0.7″ N 80°42′17.31″ E), Deniyaya (6°20′32.95″ N 80°33′34.77″ E), and Sinharaja (6°23′23″ N 80°30′5″ E) (Figure 1). The study permit was obtained by the Department of Wildlife Conservation in Sri Lanka (permit no. WL/3/2/62/21), and ethical consent for blood collection was obtained from the Institute of Biology in Sri Lanka (ERC no: 267/06/2021). The blood samples (approximately 2 mL) were collected by a veterinary surgeon using ethylenediaminetetraacetic acid (EDTA) tubes (to prevent coagulation). Subsequently, the samples were stored at −13 °C until DNA extraction was conducted. Glassware and stock solutions underwent routine sterilization by autoclave (TOMY Seiko Co., Ltd. ES-315, 3-14-17 Tagara, Tokyo 179-0073, Japan) for 15 min at 120 °C. The sample collection was carried out in December of 2021 and March and May of 2022.

### 2.2. DNA Extraction, Polymerase Chain Reaction (PCR), and Sequencing

A QIAGEN DNeasy blood and tissue kit was used to extract DNA from the blood samples following the manufacturer’s protocol. A 468 bp fragment of the *COI* gene was amplified using the following primers: 5′-TTGGTGCCTGAGCAGGCATAGT-3′ and 5′-GGAACAAGTGTTGATATAGAAT-3′ [17], and a 574 bp fragment of the *Cyt b* gene was amplified using the following synthesized primers: 5′-ATGGATCTGAGGGGGCTTTT-3′ and 5′-CTAGCAATATTAAGAAGAGT-3′. All primer concentrations were one µL. Amplification was completed using a gradient (47–64 °C) PCR (Life EcoTM Gradient Thermal Cycler, Hangzhou Bioer Technology, Hangzhou, China), and negative controls were used to monitor the efficacy and reliability of the reactions. The final PCR volume was 25 μL, with each tube containing 0.25 μL of each primer, two μL of extracted DNA, 14.5 μL of PCR water, and 0.3 μL of Taq polymerase (2 U/μL) (Promega Inc., Madison WI, USA). The PCR products were resolved by gel electrophoresis through a 1.5% agarose gel and visualized by a UV transilluminator (IGene Labserve Private Limited, Ashok Nagar, New Delhi, India). The optimized PCR conditions were as follows: an initial denaturation at 94 °C for 2 min, followed by 40 cycles of denaturation at 92 °C for 30 s, annealing at 50 °C for 30 s, and extension at 72 °C for 30 s, with a final extension step of 72 °C for 15 min, which was used for the *COI* region. Similarly, for the *COI* region, we employed an initial denaturation at 94 °C for 3 min, followed by 35 cycles of denaturation at 94 °C for 30 s, annealing at 60 °C for 30 s, and extension at 72 °C for 30 s, and a final extension step of 72 °C for 10 min was adopted. All the succeeded, unpurified PCR amplicons were sent to Macrogen (Seoul, Republic of Korea) for sequencing. 

For each PCR product, sequences were obtained for the forward and reverse direction and aligned using Geneious Prime 2020.0.5, and the consensus sequences were extracted for analysis. Initially, the consensus sequence of each Sambar sample was aligned with NCBI database sequences using the essential local alignment search tool (BLAST) to identify the individual species and to obtain the highest similarity to the query sequence.

### 2.3. Phylogenetic Analyses and Divergence Times

Multiple sequence alignments were conducted for each gene region, and we combined the regions using the ClustalW tool (MEGA 7.0 version) [18]. The best partition schemes and evolutionary models were selected for the Bayesian analysis using PartitionFinder v. 2.1.1 [19]. A Bayesian tree was generated with posterior probability values for the combined alignment of the *Cyt b* and *COI* regions using MrBayes v.3.2.7 [20]. Four Metropolis-coupled Markov chain Monte Carlo (MCMC) were run for 10,000,000 generations and the trees were sampled every 1000 generations. An initial 25% of the sampled trees were discarded as they were from the burn-in period, and the consensus tree was checked using FigTree.v.1.4.4. The tree was rooted using *Muntiacus reevesi* and *Muntiacus crinifrons* as the outgroups. Pairwise genetic distances were calculated using the Kimura two-parameter model by Mega 7.

Molecular divergence time estimations were performed in BEAST v.1.8.4 [21] on the concatenated *Cyt b* and *COI* sequences. Since similar results were obtained for both analyses (MrBayes and BEAST), we further validated and confirmed the consistency of the results.

The TN93 model used as the best-fit model of nucleotide substitution for both partitions (*Cyt b* and *COI*), as suggested by PartitionFinder v. 2.1.1. The uncorrected log-normal relaxed clock was used as the clock model, and we used the “Yule species tree prior model” [22]. To calibrate the divergence times on the phylogenetic tree against the fossil records, we specified a normal prior distribution of 8  ±  1 million years ago (MYA) for the node of Cervinae and the Muntiacinae subfamily [23]. Two independent runs with Markov chain Monte Carlo (MCMC) chain lengths of 50 million generations were executed in BEAST v.1.8.4, and the trees were sampled every 1000 generations. Convergence of the two parallel runs was assessed in Tracer v.1.7.1 [24] by checking the effective sample sizes (ESSs) of the parameters (values of >200). The samples collected during the initial burn-in (10%) were discarded. The maximum credibility clades of the tree were created using TreeAnnonator v.1.8.4. All topologies were edited and visualized by the software FIGTREE v.1.4.4.

The Lineages Through Time plot (LTT plot) [25] was constructed by plotting the log-transformed number of lineages against the age of the node (from the Bayesian chronogram). The LTT plot was created using R package, excluding the outgroups.

## 3. Results

### 3.1. Phylogenetic Analysis

**Figure 2:**
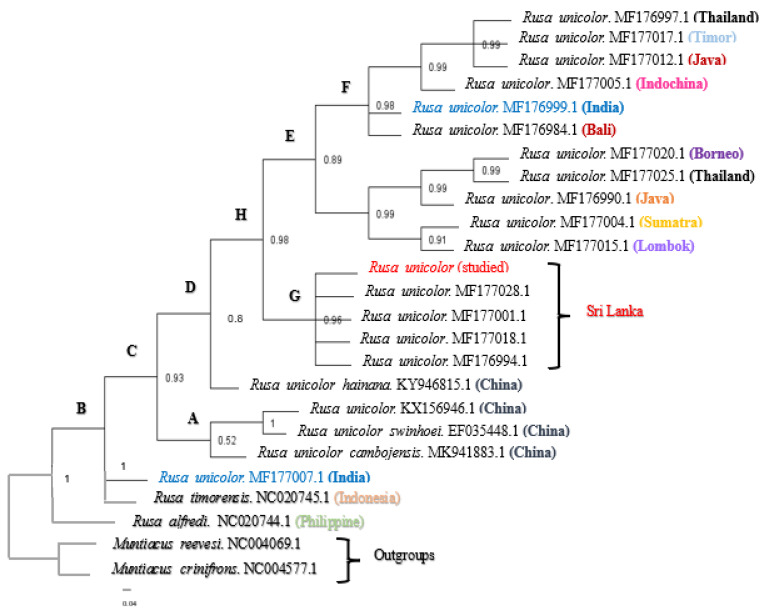
The Bayesian phylogenetic tree inferred from the mitochondrial *Cyt b* and *COI* gene sequences from *Rusa unicolor*. The numbers represent the posterior probability values estimated by MrBayes (MB). The nodes with posterior probability values of less than 0.50 were absent. (A–H nodes are used for the explaining purposes).

The results of the Bayesian analysis indicated that the branches and clusters in the phylogenetic trees were statistically verified. According to one of the phylogenetic trees (Figure 2), we confirmed that the phylogeny of the Sri Lankan Sambar deer sequence was clustered in one clade (clade G), namely, *Rusa unicolor*, with a posterior probability (pp) value of 0.96. However, the Indian Sambar deer was paraphyletic and found in two major distinct cades. The first, clade F, included *Rusa unicolor* from India (MF176999.1), Bali (MF176984.1), Indochina (MF177005.1), Java (MF177012.1), Timor (MF177017.1), and Thailand (MF176997.1), and it had a probability value of 0.98. The second, clade B, included *Rusa unicolor* (MF177007.1) from India.

According to Figure 2, the Sambar deer in India and Bali indeed represent species distinct from the Sambar deer in Indochina, Java, Timor, and Thailand. On the contrary, the Sambar deer from Bali were the most closely related species to the Indian Sambar deer (MF176999.1). All the Sambar deer species in China, including two subspecies of *Rusa unicolor* (*Rusa unicolor swinhoei* and *Rusa unicolor cambojensis*), were clustered in clade A, except for the subspecies *Rusa unicolor hainana,* which was clustered as a separate node. However, clade A exhibited a probability value of 0.52, indicating that the node was represented as a polytomy. 

### 3.2. Genetic Distances

Pairwise genetic distances were obtained for the combined sequences (*COI* and *Cyt b*) used for constructing the phylogenetic tree using the Kimura two-parameter model in Mega 7. The results are shown in Table 1.

The pairwise distances of the Sri Lankan Sambar deer species (MF177028.1, MF177001.1, MF177018.1, and MF176994.1) varied between 0.2491 and 0.2495. The minimum pairwise distance was obtained for the currently studied species. This genetic distance further confirmed the result of the phylogenetic analysis, indicating a close relationship between the studied Sambar deer and other Sri Lankan Sambar deer. 

Relevant pairwise genetic distances among the taxa of the clades also supported all of the monophyletic clades and the sister taxa formed from the phylogenetic analysis. The genetic distances of two (MF176999.1 and MF177007.1) Indian Sambar deer (0.2531 and 0.2541, respectively) were comparatively higher than those of the Sri Lankan Sambar deer. The genetic distances of *Rusa timorensis* and *Rusa alfredi* were 0.2539 and 0.2550, respectively. However, the highest genetic distance values of 2.7543 and 2.9050 were obtained for the outgroups (NC004069.1 and NC004577.1, respectively).

### 3.3. Divergence Times

**Figure 3:**
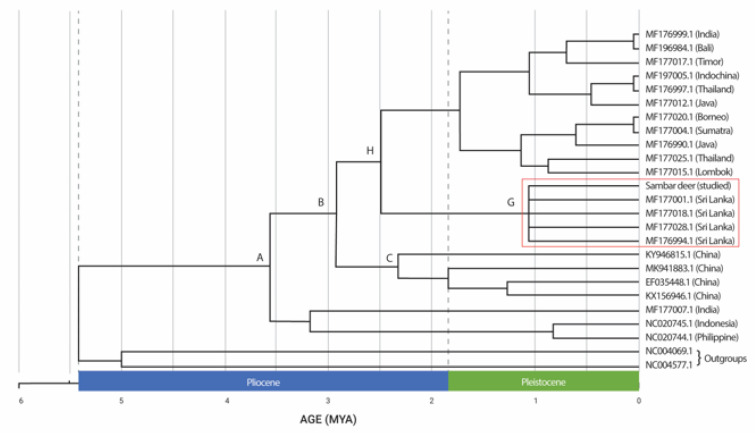
Bayesian chronogram for *Rusa unicolor* (million years ago (MYA). Sri Lankan *Rusa unicolor* is denoted in the red box.

The divergence time estimations based on the concatenated sequences of *Cyt b* and *COI* showed a strongly supported time-calibrated tree (Figure 3). The earliest diversification of *Rusa unicolor* was observed in the early Pliocene era, more than 5.4 MYA. The first split occurred at approximately 3.5 MYA in the Pliocene era. The Sri Lankan Sambar deer species (MF177001.1, MF177018.1, MF177028.1, and MF176994.1), including the studied Sambar deer, diverged at approximately 1.1 MYA during the Pleistocene era, forming the sister taxa. Sambar deer from India (MF176999.1) have clustered with other Sambar deer from Bali, Timor, Indochina, Thailand, Java, Borneo, Sumatra and Lombok and they diverged during the Pleistocene era. The split between the Sambar deer in China (node C) and those in Sri Lanka (node G) occurred approximately 3.1 MYA during the Pliocene era. Hence, based on the results, we concluded that *Rusa timorensis* and *Rusa alfredi* diverged during the Pleistocene era.

**Figure 4:**
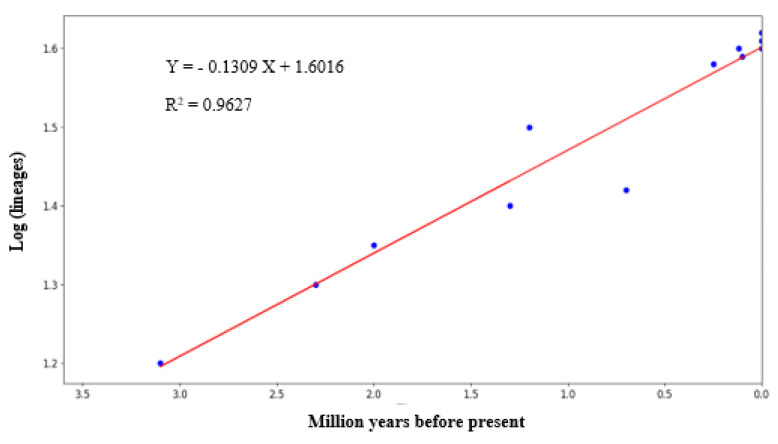
The lineage through time plot (LTT plot) of Sambar deer derived from the Bayesian chronogram of the logarithm numbers of species against the millions of years before the present.

Figure 4 depicts the graph-plotted logarithm number of species against the millions of years before the present day, as derived from the Bayesian chronogram. The relatively linear slope of the LTT plot suggested that the diversification rate of *Rusa unicolor* was seen for millions of years. According to the graph, the earliest diversification among Sambar deer dated back to 3.15 MYA in the Middle Pliocene era.

## 4. Discussion

In mammals, maternal inherited DNA does not undergo recombination, resulting in individuals having a single DNA sequence [26]. It has been revealed that mitochondrial DNA is a valuable tool for studying population genetics and molecular phylogenetics [27]. Mitochondrial DNA (mtDNA) possesses several favorable characteristics, including that it is present in large quantities, has a small genome size, and has extremely low probability. Further, mtDNA exhibits higher mutation rates than nuclear DNA, which changes only through mutations. Higher mutation rates produce higher degrees of sequence variations, which makes mtDNA a potentially useful biomarker for phylogenetic studies. These features have made mtDNA one of the most frequently used markers in molecular systematics. Hence, in this study, we used the mtDNA markers *COI* and *Cyt b* for the taxonomic identification of the studied subject, *Rusa unicolor* [28,29]. Further, we determined the evolutionary divergence of the Sri Lankan Sambar deer. 

In the BI (Bayesian Inference) analysis, the pp values higher than 0.8 in most of the branches indicated the reliability of the estimated phylogenetic relationship among the Sambar deer species worldwide. The posterior probability values of all the nodes except node A (pp = 0.52) were nearly one, which indicated that there were well-supported branches for most of the clades [30]. The short branching found in node A may have been due to variable sites among the individuals within the species. Sri Lankan Sambar deer, including the studied Sambar deer, formed a monophyly (pp = 0.96) in the BI tree. In addition, when considering the pairwise genetic distances, the least pairwise distance was obtained for the other Sri Lankan Sambar deer when compared to the studied Sambar deer. The genetic distance of the Sri Lankan Sambar deer was further confirmed by the monophyly of clade G. The distinct Sri Lankan clade G provided additional evidence for recognizing the Sri Lankan Sambar deer as unique. This support came from the karyotype differences (2n = 56 in the Sri Lankan Sambars, 2n = 58 in the Indian Sambars, and 2n = 62 in the Chinese and Malaysian Sambars) and morphological assessments [1]. Furthermore, clade F (pp = 0.98), clade E (pp = 0.89), clade H (pp = 0.98), and clade C (pp = 0.93) were observed to be monophyletic. 

The phylogenetic analysis did not support the monophyly of the Indian and Sri Lankan Sambars, which means they are not genetically similar to each other (genetic variations can be seen in the entire genome), and there was a significant pairwise genetic distance between the Indian and the Sri Lankan Sambar deer. The fact that there are Indian haplotypes at the base of the Sambar deer clade (MF177007.1) and nested within that clade close to Timor and Bali (MF176999.1) suggests that there is a large variability within the Indian Sambar deer. Although the most recent ancestor is not that distant in evolutionary times, the results emphasized the formation of a subspecies. It has been argued that the differentiation of the Sri Lankan population of Sambar deer from the Indian population is the result of their isolation in the wet zones of Sri Lanka during the Pleistocene glacial periods. The phylogeny did not support the monophyly of the Indian Sambar deer in which they are clustered with Sambar deer from other Asian countries. These results indicate that the Indian Sambar deer population is genetically mixed. The reason may be that they constitute a large population as they exhibit a greater number of genetic variations such as mutations resulting from coexistence and competition within species [31]. The studied Sambar deer did not closely relate to the *Rusa unicolor* subspecies in China, which may be because the current study was limited to DNA barcodes instead of considering the entire genome. 

According to the phylogeographic data, the present study did not support separating the clades and subclades into distinct taxonomic units. Therefore, we constructed a chronogram to clarify the results further. The chronogram’s divisions could indicate regions where Sambar distributions were concentrated. These areas might have remained unchanged during natural disasters and man-made influences. The branching order of clade B indicated colonization from Northern Indochina southwards to Sri Lanka. However, species that retained broad ecological niches, such as Sambar deer, could have utilized newly emerged habitats. Such scenarios could cause the different haplotypic distribution patterns observed here.

The Sri Lankan Sambar deer is suggested to have diverged during the Pleistocene era, coinciding with the timespan of 1.1 MYA. During the Pleistocene epoch, the Sri Lankan island was part of mainland India. Due to the cold temperatures of the Pleistocene era, the scattered glaciers caused the water level to fall, resulting in the inundation of many places [32]. The cooling and drying of the global environment may have contributed to the enormous spread of grasslands and savannas during this time. The change in vegetation may be a significant factor in the rise of grazers such as the Sambars who came to live in these areas.

To study the diversification rate of lineages within Sambar deer, we employed the Lineages Through Time (LTT) plot approach [33]. The pattern observed in the LTT plot in the present study showed a linear increment. According to previous studies, for a typically sized clade, an LTT plot would exhibit a straight line on a semi-log plot [34]. Hence, the linear slope observed in this study suggested that the diversification rate of *Rusa unicolor* decreased with time.

The primary aim of this study was to identify informative molecular markers to distinguish Sri Lankan *Rusa unicolor* species from other Sambar deer species. The present study also provided evidence that mtDNA *Cyt b* and *COI* would be effective for identifying Sambar deer species and analyzing their genetic relationships. As the Sri Lankan Sambar deer was identified only up to the species level, further studies need to be conducted to substantiate the occurrence in the subspecies level. The morphological and molecular data will provide a steady platform for identifying Sambar deer worldwide. Furthermore, DNA barcode information can be added to new species descriptions and taxonomic revisions. DNA barcodes offer a valuable understanding of intraspecific variation and specialized knowledge of the evolutionary connections between these species. 

## 5. Conclusions

Collectively, the results demonstrated the effectiveness of mtDNA and, in particular, *Cyt b* and *COI* sequences for detecting species-level differences in *Rusa unicolor*. The phylogenetic position revealed that it could be a genetically different species from the Indian *Rusa unicolor* and other *Rusa unicolor* subspecies. The gradual difference in Peninsular India post-Pleistocene era approximately 1.1 MYA may have been a momentous event that led to a divergence in the Sri Lankan Sambar deer.

## Figures and Tables

**Table 1:** Pairwise genetic distance comparison of the Sri Lankan Sambar deer. Sri Lankan Sambar species are highlighted in yellow color.

		Origin	1	2	3	4	5	6	7	8	9	10	11	12	13	14	15	16	17	18	19	20	21	22	23	24
1	Studied sambar deer	Sri Lanka																								
2	MK941883.1	China	0.2548																							
3	KY946815.1	China	0.2538	0.0072																						
4	EF035448.1	China	0.2532	0.0072	0.0062																					
5	MF176999.1	India	0.2531	0.0109	0.0088	0.0098																				
6	MF176994.1	Sri Lanka	0.2495	0.0145	0.0124	0.0135	0.0109																			
7	MF177018.1	Sri Lanka	0.2495	0.0145	0.0124	0.0135	0.0109	0.0000																		
8	MF177001.1	Sri Lanka	0.2492	0.0140	0.0119	0.0130	0.0103	0.0005	0.0005																	
9	MF177028.1	Sri Lanka	0.2491	0.0140	0.0119	0.0130	0.0103	0.0005	0.0005	0.0000																
10	MF177015.1	Lombok	0.2531	0.0120	0.0093	0.0114	0.0057	0.0125	0.0125	0.0120	0.0120															
11	MF177012.1	Java	0.2485	0.0121	0.0089	0.0110	0.0036	0.0115	0.0115	0.0110	0.0110	0.0052														
12	MF177007.1	India	0.2541	0.0098	0.0088	0.0088	0.0103	0.0140	0.0140	0.0135	0.0135	0.0130	0.0115													
13	MF177004.1	Sumatra	0.2538	0.0130	0.0103	0.0124	0.0046	0.0135	0.0135	0.0130	0.0130	0.0041	0.0042	0.0130												
14	MF177025.1	Thailand	0.2531	0.0103	0.0072	0.0093	0.0036	0.0114	0.0114	0.0109	0.0109	0.0021	0.0031	0.0109	0.0041											
15	MF177017.1	Timor	0.2534	0.0124	0.0098	0.0109	0.0051	0.0140	0.0140	0.0135	0.0135	0.0026	0.0047	0.0135	0.0036	0.0026										
16	MF177020.1	Borneo	0.2541	0.0130	0.0093	0.0124	0.0046	0.0135	0.0135	0.0130	0.0130	0.0052	0.0021	0.0130	0.0041	0.0041	0.0046									
17	MF176997.1	Thailand	0.2528	0.0124	0.0103	0.0114	0.0015	0.0124	0.0124	0.0119	0.0119	0.0073	0.0052	0.0119	0.0062	0.0051	0.0067	0.0062								
18	MF177005.1	Indochina	0.2518	0.0111	0.0090	0.0100	0.0005	0.0111	0.0111	0.0106	0.0106	0.0063	0.0042	0.0106	0.0053	0.0042	0.0058	0.0053	0.0010							
19	NC004069.1	China	2.7543	0.1184	0.1172	0.1159	0.1153	0.1165	0.1165	0.1171	0.1171	0.1191	0.1162	0.1191	0.1146	0.1172	0.1165	0.1146	0.1146	0.1168						
20	NC004577.1	China	2.9050	0.1180	0.1168	0.1155	0.1136	0.1187	0.1187	0.1193	0.1193	0.1193	0.1171	0.1149	0.1168	0.1168	0.1174	0.1168	0.1130	0.1145	0.0646					
21	KX156946.1	China	0.2541	0.0072	0.0051	0.0021	0.0098	0.0135	0.0135	0.0130	0.0130	0.0114	0.0099	0.0088	0.0124	0.0093	0.0119	0.0114	0.0114	0.0100	0.1159	0.1168				
22	MF176990.1	Java	0.2533	0.0124	0.0088	0.0119	0.0062	0.0119	0.0119	0.0114	0.0114	0.0067	0.0036	0.0124	0.0057	0.0057	0.0062	0.0026	0.0077	0.0063	0.1141	0.1162	0.0109			
23	MF176984.1	Bali	0.2541	0.0135	0.0098	0.0130	0.0051	0.0140	0.0140	0.0135	0.0135	0.0057	0.0026	0.0135	0.0046	0.0046	0.0051	0.0005	0.0067	0.0058	0.1140	0.1161	0.0119	0.0021		
24	NC020745.1	Indonesia	0.2539	0.0070	0.0068	0.0072	0.0105	0.0088	0.0088	0.0085	0.0085	0.0110	0.0108	0.0115	0.0140	0.0113	0.0134	0.0140	0.0127	0.1194	0.119	0.0070	0.0128	0.0139	0.0067	
25	NC020744.1	Philippine	0.255	0.0083	0.0072	0.0092	0.0110	0.0092	0.0092	0.0090	0.0090	0.0120	0.0115	0.0125	0.0155	0.0115	0.0140	0.0147	0.0130	0.1198	0.1205	0.0075	0.0133	0.0143	0.0072	0.0054

## Data Availability

All the data are included in the manuscript.
